# No association of DRD2, DRD3, and tyrosine hydroxylase gene polymorphisms with personality traits in the Japanese population

**DOI:** 10.1186/1744-9081-2-32

**Published:** 2006-10-03

**Authors:** Hiroyuki Hibino, Mamoru Tochigi, Takeshi Otowa, Nobumasa Kato, Tsukasa Sasaki

**Affiliations:** 1 Department of Neuropsychiatry, Graduate School of Medicine, University of Tokyo, Tokyo, Japan; 2 Health Service Center, University of Tokyo, Tokyo, Japan

## Abstract

**Background:**

Dopamine D2 receptor (*DRD2*) and dopamine D3 receptor (*DRD3*) genes could be candidates for personality-related genes considering their pharmacological profiles or structures. However, a limited number of studies have investigated the association between these genes and personality traits. In the present study, we investigated the *DRD2, DRD3*, and tyrosine hydroxylase (*TH*) genes in relation to personality traits in the Japanese population. Epistasis (gene-gene interaction) among the genes was extensively analyzed, in addition to the analysis based on each gene.

**Methods:**

The -241A/G, -141C Ins/Del, and Ser311Cys polymorphisms in the *DRD2 *gene, the Ser9Gly polymorphism of the *DRD3 *gene, and the Val81Met and PstI site polymorphisms in the *TH *gene were genotyped in 257 healthy Japanese subjects. Personality traits were evaluated by using the Revised NEO Personality Inventory (NEO PI-R) and the State-Trait Anxiety Inventory (STAI). The associations between gene polymorphisms and the scores for NEO PI-R or Trait Anxiety of STAI were statistically analyzed by one-way analysis of covariance (ANCOVA) adjusting sex and age. Epistasis was assessed using two-way ANCOVA between the polymorphisms of independent two genes.

**Results:**

In the analysis based on each gene, trends for association were observed between State Anxiety and the *DRD2 *-141C Ins/Del polymorphism (p = 0.031, uncorrected), and between Trait Anxiety and the *DRD2 *Ser311Cys or *TH *PstI site polymorphism (p = 0.048 and 0.041, respectively, uncorrected). In epistatic analysis, a trend for interaction was observed on the scores for Neuroticism and Trait Anxiety between the *DRD2 *-141C Ins/Del and *TH *Val81Met polymorphisms (p = 0.015 and 0.010, respectively, uncorrected). However, these differences were insignificant after Bonferroni correction.

**Conclusion:**

The present study did not provide evidence for the association between these dopamine-related genes and personality traits in the Japanese population.

## Background

Dopamine is one of the neurotransmitters, which affect various mental functions and behaviors. Pharmacological research on schizophrenia has proposed the dopamine hypothesis, which states that symptoms of schizophrenia result from excessive dopaminergic activity [1]. Personality traits have also been considered to depend on the secretion and metabolism of dopamine [2]. Based on this concept, several molecular genetic studies have been conducted on personality traits with respect to dopamine-related genes. Particularly, the association between a 48 bp variable number tandem repeat (VNTR) polymorphism in exon 3 of the dopamine D4 receptor (*DRD4*) gene and novelty seeking (NS) has been studied intensively ever since the first study conducted by Ebstein et al. [3]. However, a limited number of studies have investigated other dopamine receptor genes, such as dopamine D2 receptor (*DRD2*) and dopamine D3 receptor (*DRD3*) genes. The DRD4 structurally resembles the DRD2 and DRD3 and displays a pharmacological profile similar to that of the DRD2. In addition, the abundance of DRD4 is extremely low compared with DRD2 [4]. Therefore, the *DRD2 *and *DRD3 *genes could also be candidates for personality-related genes. In the present study, we investigated the *DRD2, DRD3*, and tyrosine hydroxylase (*TH*) genes, which is the rate-limiting enzyme involved in the synthesis of catecholamines, in relation to personality traits in Japanese subjects. In addition to the association of each gene, epistasis (gene-gene interaction) among the genes was also assessed.

In the field of neuroimaging, a relation between the density of the DRD2 and the personality trait of novelty seeking (NS) was indicated [5]. A significant correlation between DRD2 density in the brain and a detached personality was also observed by Farde et al. [6], which was replicated by Breier et al. [7]. In the molecular genetic study of personality traits, TaqI A polymorphism in the 3' region was the first focus of attention, although the polymorphism is not functional. The association between TaqI A1 allele and NS has been mainly investigated [8]. Several functional polymorphisms have been studied in the *DRD2 *gene. The Ser311Cys polymorphism is a missense mutation located in exon 7, the putative third cytoplasmic loop. The Cys allele was shown to be less effective than the Ser allele in inhibiting cAMP synthesis [9], indicating a functional deficit of the Cys allele. However, no association was observed between the polymorphism and personality traits in Gebhardt et al. [10]. To our knowledge, no other studies investigated the association between the Ser311Cys polymorphism and personality traits. The -141C Ins/Del polymorphism, located in the promoter region, was observed to affect luciferase activity in vitro [11]. The polymorphism was also observed to be in linkage disequilibrium with the functional C957T polymorphism [12]. Jonsson et al. [13] observed the association between the -141C Del variant and a detached personality, while Katsuragi et al. [14] did not observe any association between the polymorphism and personality traits in the Japanese subjects. The -241A/G polymorphism is not functional and has no effect on luciferase activity in vitro [11]. To our knowledge, no association study has been conducted between the polymorphism and personality traits.

With respect to the *DRD3 *gene, the Ser9Gly polymorphism was investigated in relevant to personality traits. Genotypes of this variant were observed to affect their dopamine binding affinity [15], indicating a possible functional effect. Czermak et al. [16] observed the association between DRD3 mRNA expression in peripheral blood lymphocytes and the personality trait of persistence. Staner et al. [17] observed the association between the polymorphism and NS in bipolar patients. While Ebstein et al. [18] observed a significant interaction among the *DRD3, DRD4*, and serotonin 2C receptor (*5-HT*_*2C*_) gene polymorphisms on reward dependence, no association was found between the polymorphism and personality traits in healthy subjects [19,20]. To our knowledge, no association study has been conducted between the *DRD3 *gene and personality traits in Japanese subjects.

Another candidate, TH is the rate-limiting enzyme involved in the synthesis of catecholamines; it converts tyrosine to dihydroxyphenylalanine (DOPA). A (TCAT)_n _repeat polymorphism in the first intron, which was suggested to have a relationship with catecholamine turnover rates [21], was investigated in relation to personality traits [22]. A tendency for high score for Neuroticism was observed in carriers of T8 allele, and high score for Conscientiousness and low score for Openness in women with T6/T10 genotype. No other polymorphism within this gene was investigated, including Val81Met in exon 3 [23] or PstI site in exon 10 [24], in relation to personality traits. Thus, further investigation will be needed to clarify the relationships between the *DRD2, DRD3*, and *TH *genes and personality traits.

## Subjects and Methods

The subjects included 257 unrelated healthy volunteers (65 males and 192 females; age, 37.3 ± 11.9 years (mean ± SD)) recruited from the staff of several mental and general hospitals around Tokyo. They had no history of major psychiatric illness. The research protocol was approved by the ethics committee of the University of Tokyo. Written informed consent was obtained from all the subjects.

After blood collection, the subjects filled out the Revised NEO Personality Inventory (NEO PI-R), a self-report inventory based on the five-factor model of personality: Neuroticism, Extraversion, Openness to Experience, Agreeableness, and Conscientiousness [25]. To further investigate the relation with anxiety, the State-Trait Anxiety Inventory (STAI), an instrument for measuring anxiety differentiating between the temporary condition of State Anxiety and the more general and long-standing quality of Trait Anxiety [26], was also completed by the subjects. Genomic DNA was extracted from leukocytes by using a standard method. The -241A/G, -141C Ins/Del, and Ser311Cys polymorphisms in the *DRD2 *gene, the Ser9Gly polymorphism of the *DRD3 *gene, and the Val81Met and PstI site polymorphisms in the *TH *gene were genotyped. Genotyping of the -241A/G and -141C Ins/Del polymorphisms of the *DRD2 *gene was performed as described by Arinami et al. [11]; Ser311Cys of the *DRD2 *gene by Higuchi et al. [27]; the *DRD3 *gene by Ebstein et al. [18]; the Val81Met of the *TH *gene by Ishiguro et al. [23]; and the PstI site of the *TH *gene by Furlong et al. [24].

The associations between gene polymorphisms and the scores for NEO PI-R or STAI were statistically analyzed by one-way analysis of covariance (ANCOVA) adjusting sex and age. Epistasis was assessed using two-way ANCOVA adjusting sex and age between the polymorphisms of independent two genes. Independence between the genes was assessed by means of chi-square test in contingency tables with marginals defined by genotype counts. Bonferroni correction was conducted for multiple testing. The statistical package SPSS for Windows [28] was used for all analyses.

## Results

Table 1 summarizes genotypic distributions of the polymorphisms in the three genes and the scores for NEO PI-R and STAI according to the genotypes. The distributions of all six polymorphisms follow the Hardy-Weinberg equilibrium. In the analysis based on each polymorphism, one-way ANCOVA showed significant difference in the score for State Anxiety according to the genotype of the -141C Ins/Del polymorphism in the *DRD2 *gene (F = 3.53, df = 2, p = 0.031); That for Trait Anxiety, according to the genotype of the Ser311Cys polymorphism in the *DRD2 *gene or the PstI site polymorphism in the *TH *gene (F = 3.96, df = 2, p = 0.048 and F = 3.25, df = 2, p = 0.041, respectively). However, statistical levels of these differences became insignificant after Bonferroni correction. No other significant difference in the scores for the NEO PI-R or STAI factors was observed in this comparison.

**Table 1:** The scores for NEO PI-R and STAI according to the genotypes of each polymorphism

	NEO PI-R factors						STAI		
		
Genotype	N (%)	Neuroticism	Extraversion	Openness	Agreeableness	Conscientiousness	N (%)	State Anxiety	Trait Anxiety
DRD2 gene									
									
-241A/G polymorphism									
A/A	197 (77.9)	100.4 ± 20.7	97.3 ± 15.1	110.5 ± 14.3	112.9 ± 12.7	100.6 ± 16.6	184 (79.3)	42.3 ± 9.7	44.6 ± 10.7
A/G	53 (20.9)	94.6 ± 18.6	99.4 ± 12.3	108.7 ± 12.7	115.2 ± 14.3	102.6 ± 18.0	45 (19.4)	38.8 ± 7.5	39.8 ± 8.9
G/G	3 (1.2)	110.0 ± 17.1	98.0 ± 15.1	112.7 ± 17.7	120.3 ± 3.1	105.3 ± 12.7	3 (1.3)	42.7 ± 7.5	45.3 ± 7.6
									
-141C Ins/Del polymorphism									
Ins/Ins	159 (62.6)	98.0 ± 20.0	97.3 ± 14.2	109.3 ± 14.2	113.7 ± 12.4	102.0 ± 16.8	146 (62.7)	42.7 ± 9.6*	43.8 ± 10.5
Ins/Del	83 (32.7)	101.4 ± 19.1	99.2 ± 15.0	111.5 ± 13.8	112.3 ± 14.5	99.4 ± 17.1	76 (32.6)	39.8 ± 8.3	43.3 ± 10.3
Del/Del	12 (4.7)	104.4 ± 25.1	92.7 ± 16.0	111.8 ± 10.5	119.9 ± 7.9	99.2 ± 15.7	11 (4.7)	39.3 ± 11.8	45.7 ± 11.8
									
Ser311Cys polymorphism									
Ser/Ser	240 (94.5)	99.6 ± 20.2	97.9 ± 14.6	110.5 ± 13.8	113.4 ± 13.1	100.7 ± 16.9	222 (94.9)	41.8 ± 9.4	44.0 ± 10.5*
Ser/Cys	14 (5.5)	90.1 ± 11.8	96.3 ± 12.9	104.4 ± 14.7	117.7 ± 10.5	107.0 ± 9.9	12 (5.1)	37.4 ± 7.9	37.1 ± 5.6
									
Ser9Gly polymorphism of the DRD3 gene									
Ser/Ser	125 (52.5)	99.8 ± 19.8	98.3 ± 15.3	110.4 ± 14.2	113.3 ± 13.3	101.5 ± 17.2	116 (53.2)	41.3 ± 8.4	43.4 ± 9.4
Ser/Gly	92 (38.7)	97.3 ± 19.8	97.7 ± 13.1	110.8 ± 13.8	114.1 ± 12.4	101.9 ± 17.1	82 (37.6)	41.0 ± 10.1	42.6 ± 11.2
Gly/Gly	21 (8.8)	103.3 ± 19.4	95.4 ± 15.4	106.9 ± 14.0	112.0 ± 15.0	93.5 ± 13.7	20 (9.2)	44.7 ± 9.9	48.0 ± 12.5
									
TH gene									
									
Val81Met polymorphism									
Val/Val	24 (12.4)	96.5 ± 22.6	101.0 ± 17.0	108.2 ± 16.5	110.8 ± 8.8	98.7 ± 17.0	22 (12.4)	42.0 ± 8.7	44.2 ± 10.4
Val/Met	80 (41.2)	100.2 ± 17.5	96.1 ± 14.4	108.4 ± 15.2	115.2 ± 12.3	100.7 ± 16.7	74 (41.8)	41.3 ± 7.5	45.1 ± 10.0
Met/Met	90 (46.4)	100.1 ± 20.9	98.7 ± 13.6	111.0 ± 12.4	112.3 ± 12.8	98.7 ± 17.3	81 (45.8)	40.8 ± 10.1	42.3 ± 10.5
									
Pst I site polymorphism									
AA	43 (18.4)	96.2 ± 20.8	100.6 ± 14.4	112.5 ± 11.6	112.2 ± 12.1	99.9 ± 17.1	42 (19.0)	40.6 ± 9.8	41.6 ± 11.1*
AB	130 (55.5)	100.8 ± 19.8	96.1 ± 14.5	109.3 ± 14.1	113.9 ± 14.0	100.6 ± 16.4	122 (55.2)	41.8 ± 9.4	44.9 ± 10.3
BB	61 (26.1)	96.9 ± 18.7	101.0 ± 14.2	109.6 ± 14.5	114.1 ± 9.8	101.4 ± 17.4	57 (25.8)	41.3 ± 8.4	42.2 ± 10.0

Scores presented as mean ± standard deviation.

* p < 0.05, ANCOVA, sex and age were included as covariates.

In epistatic analysis, no significant deviation of independence was observed for all combinations of polymorphisms in the different genes (data not shown). Two-way ANCOVA showed significant interactions on the scores for Neuroticism and Trait Anxiety between the *DRD2 *-141C Ins/Del and *TH *Val81Met polymorphisms (F = 3.18, df = 4, p = 0.015 and F = 3.43, df = 4, p = 0.010, respectively). However, statistical level of this interaction became insignificant after Bonferroni correction. No other significant interaction of the polymorphisms was observed on the NEO PI-R or STAI factors.

## Discussion

In the present study, we investigated the association of polymorphisms in the three dopamine-related genes, *DRD2, DRD3*, and *TH*, with personality traits. Epistasis among the genes was extensively analyzed, in addition to the analysis based on each gene. As a result, in the analysis based on each gene, trends for association were observed between State Anxiety and the *DRD2 *-141C Ins/Del polymorphism (p = 0.031, uncorrected), and between Trait Anxiety and the *DRD2 *Ser311Cys or *TH *PstI site polymorphism (p = 0.048 and 0.041, respectively, uncorrected). In epistatic analysis, a trend for interaction was observed on the scores for Neuroticism and Trait Anxiety between the *DRD2 *-141C Ins/Del and *TH *Val81Met polymorphisms (p = 0.015 and 0.010, respectively, uncorrected). However, these differences were insignificant after Bonferroni correction. Thus, the present study did not provide evidence for the association between these dopamine-related genes, *DRD2, DRD3*, and *TH*, and personality traits in Japanese subjects.

No association between the Ser311Cys polymorphisms in the *DRD2 *gene and personality traits in the present result was consistent with the previous study [10]. With respect to the *DRD2 *-141C Ins/Del polymorphism, the result was consistent with the previous Japanese study [14], indicating no significant association between the polymorphism and personality traits. Jonsson et al. [13] also observed no association between the polymorphism and the score for the Temperament and Character Inventory (TCI) [29] in Caucasian, although the association with detached personality was observed. The role of *DRD2 *in personality traits may be relatively small considering no association of two major functional polymorphisms. However, in the present result, the score for State or Trait Anxiety increased consecutively according to the amount of -141C Ins or 311 Ser variant, respectively. It may be worth investigating further in a larger sample, because low frequency of the -141C Del or 311 Cys variant might cause insufficient statistical power.

No significant association was observed between personality traits and *DRD3 *or *TH *polymorphisms in the present study. To our knowledge, this is the first to investigate the relation between the *TH *Val81Met or PstI site polymorphism and personality traits. With respect to the *DRD3 *Ser9Gly polymorphism, the result was consistent with the previous studies [19,20]. Ebstein et al. [18] observed a significant interaction among three gene polymorphisms, the *DRD3, DRD4*, and *5-HT*_*2C*_, on personality trait of reward dependence. In epistatic analysis of the present study, no significant interaction was observed among the genes analyzed after Bonferroni correction.

Several limitations might be considered for the present study. First is the sex unbalancing in the subjects, although sex was included as a covariate in the analyses. Second, the subjects were recruited from the hospital staff, which may not represent the general population sufficiently. Uncontrolled socio-demographic factors might affect the result. Lastly, mood fluctuation of the subjects might affect the measurement of personality traits to some extent.

## Conclusion

We provided no evidence for association between the there dopamine-related genes, *DRD2, DRD3*, and *TH*, and personality traits in Japanese subjects.

## Competing interests

The author(s) declare that they have no competing interests.

## Authors' contributions

HH and TO carried out recruitment of the subjects and the genetic studies. MT performed statistical analyses and drafted the manuscript. TS participated in the design of the study and helped to draft the manuscript. NK conceived of the study and participated in its design. All authors read and approved the final manuscript.
